# A Randomised Comparison Evaluating Changes in Bone Mineral Density in Advanced Prostate Cancer: Luteinising Hormone-releasing Hormone Agonists Versus Transdermal Oestradiol

**DOI:** 10.1016/j.eururo.2015.11.030

**Published:** 2016-06

**Authors:** Ruth E. Langley, Howard G. Kynaston, Abdulla A. Alhasso, Trinh Duong, Edgar M. Paez, Gordana Jovic, Christopher D. Scrase, Andrew Robertson, Fay Cafferty, Andrew Welland, Robin Carpenter, Lesley Honeyfield, Richard L. Abel, Michael Stone, Mahesh K.B. Parmar, Paul D. Abel

**Affiliations:** a Medical Research Council Clinical Trials Unit at University College London, London, UK; b Cardiff School of Medicine, Cardiff University, Cardiff, UK; c The Beatson West of Scotland Cancer Centre, Glasgow, UK; d Freeman Hospital, Newcastle upon Tyne, UK; e Ipswich Hospital NHS Trust, Ipswich, UK; f Scarborough General Hospital, Scarborough, UK; g Imperial College Healthcare NHS Trust, London, UK; h Imperial College London, London, UK; i Cardiff University, Bone Research Unit, Penarth, UK

**Keywords:** Androgen-deprivation therapy, Bone mineral density, Prostate cancer, Transdermal oestradiol

## Abstract

**Background:**

Luteinising hormone-releasing hormone agonists (LHRHa), used as androgen deprivation therapy (ADT) in prostate cancer (PCa) management, reduce serum oestradiol as well as testosterone, causing bone mineral density (BMD) loss. Transdermal oestradiol is a potential alternative to LHRHa.

**Objective:**

To compare BMD change in men receiving either LHRHa or oestradiol patches (OP).

**Design, setting, and participants:**

Men with locally advanced or metastatic PCa participating in the randomised UK Prostate Adenocarcinoma TransCutaneous Hormones (PATCH) trial (allocation ratio of 1:2 for LHRHa:OP, 2006–2011; 1:1, thereafter) were recruited into a BMD study (2006–2012). Dual-energy x-ray absorptiometry scans were performed at baseline, 1 yr, and 2 yr.

**Interventions:**

LHRHa as per local practice, OP (FemSeven 100 μg/24 h patches).

**Outcome measurements and statistical analysis:**

The primary outcome was 1-yr change in lumbar spine (LS) BMD from baseline compared between randomised arms using analysis of covariance.

**Results and limitations:**

A total of 74 eligible men (LHRHa 28, OP 46) participated from seven centres. Baseline clinical characteristics and 3-mo castration rates (testosterone ≤1.7 nmol/l, LHRHa 96% [26 of 27], OP 96% [43 of 45]) were similar between arms. Mean 1-yr change in LS BMD was −0.021 g/cm^3^ for patients randomised to the LHRHa arm (mean percentage change −1.4%) and +0.069 g/cm^3^ for the OP arm (+6.0%; *p* < 0.001). Similar patterns were seen in hip and total body measurements. The largest difference between arms was at 2 yr for those remaining on allocated treatment only: LS BMD mean percentage change LHRHa −3.0% and OP +7.9% (*p* < 0.001).

**Conclusions:**

Transdermal oestradiol as a single agent produces castration levels of testosterone while mitigating BMD loss. These early data provide further supporting evidence for the ongoing phase 3 trial.

**Patient summary:**

This study found that prostate cancer patients treated with transdermal oestradiol for hormonal therapy did not experience the loss in bone mineral density seen with luteinising hormone-releasing hormone agonists. Other clinical outcomes for this treatment approach are being evaluated in the ongoing PATCH trial.

**Trial registration:**

ISRCTN70406718, PATCH trial (ClinicalTrials.gov NCT00303784).

## Take Home Message

Transdermal oestradiol as a single agent produces castrate levels of testosterone in patients with prostate cancer (PCa) while avoiding the bone mineral density loss seen with luteinising hormone-releasing hormone agonists. The ongoing phase 3 PATCH trial will provide comparative data in terms of PCa efficacy and overall toxicity.

## 1 Introduction

Androgen deprivation therapy (ADT), widely used in the management of prostate cancer (PCa), is usually achieved in contemporary practice through the administration of luteinising hormone-releasing hormone agonists (LHRHa) or antagonists. More than half of the men diagnosed with PCa are expected to be treated with ADT at some point during the course of their disease, many for a decade or longer [1]. LHRHa suppress testosterone to castration levels but also deplete oestradiol because approximately 80% of oestradiol in men is derived by aromatisation from testosterone [2]. The effect of oestradiol deficiency on bone health in women is well established, but only in the last 10–15 yr has the negative impact of LHRHa on bone health in men with PCa been documented [3], [4], [5], [6].

Average declines in bone mineral density (BMD) of between 2% and 10% per year were reported in studies of men treated with LHRHa [3], [4], [5], [7], [8], resulting in an increased incidence of fractures with associated morbidity and mortality [9], [10]. The rate of fracture increases with the duration of LHRHa use as well as in men with a high baseline risk of skeletal complications [9], [10], with important implications for PCa patients who are often elderly and have other comorbidities.

Parenteral oestradiol is a potential alternative to LHRHa in the management of PCa. Administering oestradiol suppresses androgen production through a negative feedback loop involving the hypothalamic-pituitary axis [11] and avoids the fall in endogenous oestradiol associated with castration levels of testosterone. Oral oestrogen (eg, diethylstilboestrol [DES]) was previously used for ADT before the development of LHRHa but was discontinued as first-line treatment because of the mainly embolic cardiovascular (CVS) toxicity [12] that was attributed to first-pass hepatic metabolism [13]. Administration of oestradiol parenterally (eg, intravenous, intramuscular, or transdermal) avoids first-pass hepatic metabolism [14], so it should mitigate the CVS risk.

**Fig. 1:**
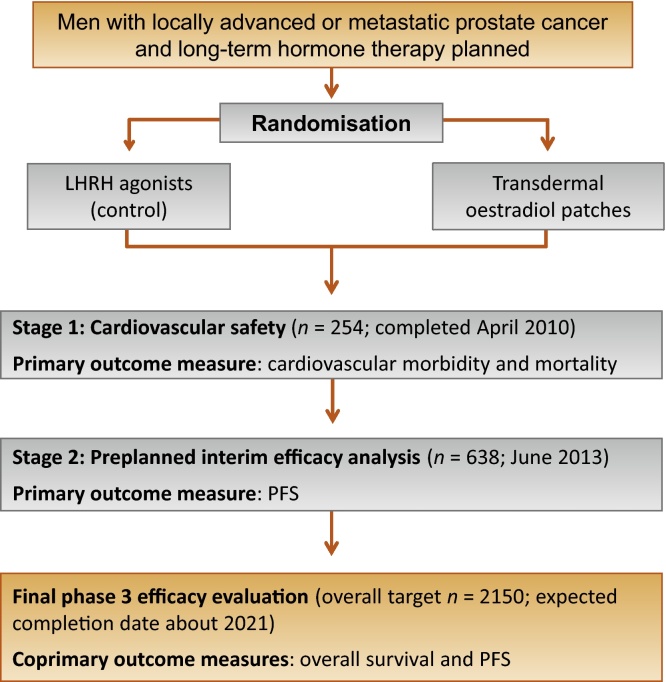
PATCH trial schema. The allocation ratio was 1:2 for luteinising hormone-releasing hormone agonists (LHRHa) to oestradiol patches during the first stage of the trial (before February 21, 2011) to optimise the experience of the patches and 1:1 thereafter. Patients in the bone mineral density study were enrolled from seven of the participating sites between August 2006 and September 2012. PFS = progression-free survival.

The ongoing randomised Prostate Adenocarcinoma TransCutaneous Hormones (PATCH) (MRC PR09) trial is assessing the safety and efficacy of transdermal oestradiol patches (OP) compared with LHRHa in the treatment of advanced PCa (Fig. 1). The first stage (*n* = 254) showed that OP produced castration levels of testosterone equivalent to LHRHa, and in addition, early CVS morbidity and mortality was similar between the two groups [15]. A further phase of recruitment included a confidential preplanned interim analysis based on progression-free survival (PFS) (*n* = 638) that led to the extension of the trial to phase 3. This is currently ongoing (target *n* = 2150), with PFS and overall survival (OS) as coprimary outcome measures.

Here we report on a preplanned study embedded within the PATCH trial comparing changes in BMD between the two hormonal treatments. It was designed to evaluate the potential benefits of transdermal oestradiol for first-line ADT in advanced PCa and to provide support for further evaluation of the clinical efficacy of this approach.

## 2 Methods

### 2.1 Study design and participants

Between August 2006 and September 2012, men who had agreed to enrol in the PATCH trial from seven preselected UK centres were approached prerandomisation for enrolment into a BMD study if they were eligible. This prospective cohort was thus a subset of the main trial population.

The study design for the main trial was previously described [15]. Briefly, men with locally advanced or metastatic PCa were eligible if they were commencing long-term (>3 yr) continuous hormonal therapy and had no history of major CVS disease. Participants were randomly allocated to receive LHRHa or transdermal oestradiol without blinding in a 1:2 ratio before February 2011 and then 1:1 (after recruitment was extended following the first stage [16]). Transdermal oestradiol was delivered, after a dose regimen change in August 2007 [16], as four FemSeven patches (100 μg/24 h) changed twice weekly during the first 4 wks. This was then reduced to three patches changed twice weekly provided testosterone levels were ≤1.7 nmol/l. LHRHa was administered as per local practice.

Patients were not eligible for the BMD study if either: their dual-energy x-ray absorptiometry (DXA) scans were likely to be nonevaluable or technically difficult due to orthopaedic prostheses or preexisting bone disease (degenerative joint disease, vertebral fractures, or bone metastases in the lumbar spine [LS]); or they were already receiving bone-strengthening agents or other medications (including calcium and vitamin D) thought to affect BMD. Patients with osteoporosis (T-score: −2.5 or lower) diagnosed on the baseline scan were also excluded.

DXA scans were performed using Lunar Prodigy (General Electric, Madison, WI, USA), Hologic Discovery, or Delphi (Hologic, Bedford, MA, USA) machines at baseline, 1 yr, and 2 yr, with the same type of machine used during follow-up. Assessments of BMD at the LS (L1–4), right hip, left hip, and whole body were made. Scans were not centrally reviewed.

The protocol was approved by national regulatory and ethics committees, and participating hospitals obtained the appropriate local approvals. Participants provided written informed consent. The Independent Data Monitoring Committee (IDMC) has permitted the release of data from the BMD study while the main trial is ongoing.

### 2.2 Statistical analysis

The primary outcome measure for the BMD study was change in LS BMD (mean L1–4) at 1 yr from baseline. Secondary outcomes were BMD change at other sites at 1 yr and changes at all sites at 2 yr.

BMD scores were compared between randomised arms using analysis of covariance, adjusting for baseline BMD. To account for different types of DXA machines used, the fitted models also included machine type and an interaction between baseline BMD and machine type, as previously done [17]. The difference between arms was estimated from the models based on absolute BMD score, then converted to percentage change (for ease of clinical interpretation) by dividing by the overall mean baseline score [18].

The target sample size was 75, allowing for 35% of patients not having a 1-yr scan (due to illness, death, or other reason). This would provide 80% power with a two-sided significance level of 0.05 to detect a difference in LS BMD change between arms, assuming the mean 1-yr change in LS BMD was −3% in the LHRHa arm and +1% in patch arm, and the standard deviation of the change from baseline was 6% [8], [17].

The primary analysis of 1-yr LS BMD change included participants enrolled after the patch dose regimen change [16] who had two or more evaluable LS vertebrae within L1–4 for both baseline and 1-yr scans. Comparison between randomised arms was based on the original treatment allocation, ignoring subsequent changes in therapy. To account for men allocated to OP but changed to LHRHa when disease progressed (permitted in the protocol), sensitivity analyses were performed including only those still on the original allocated treatment without additional systemic anticancer therapy at their 1 and 2 yr scans; men on OP with oestradiol levels <250 pmol/l at follow-up scans were considered not adhering to the patch regimen and therefore excluded. The following prespecified subgroup analyses were also performed: no bone metastases at baseline (because PCa bone metastases are predominantly osteoblastic and thus associated with increased BMD [19]); men scanned using Hologic Discovery, the most commonly used machine in the study; and for analysis of LS BMD, those with all four L1–4 vertebrae evaluable at both baseline and follow-up scans (because BMD varies with LS vertebrae).

Statistical analyses were performed using Stata v.13 (StataCorp, College Station, TX, USA).

## 3 Results

**Fig. 2:**
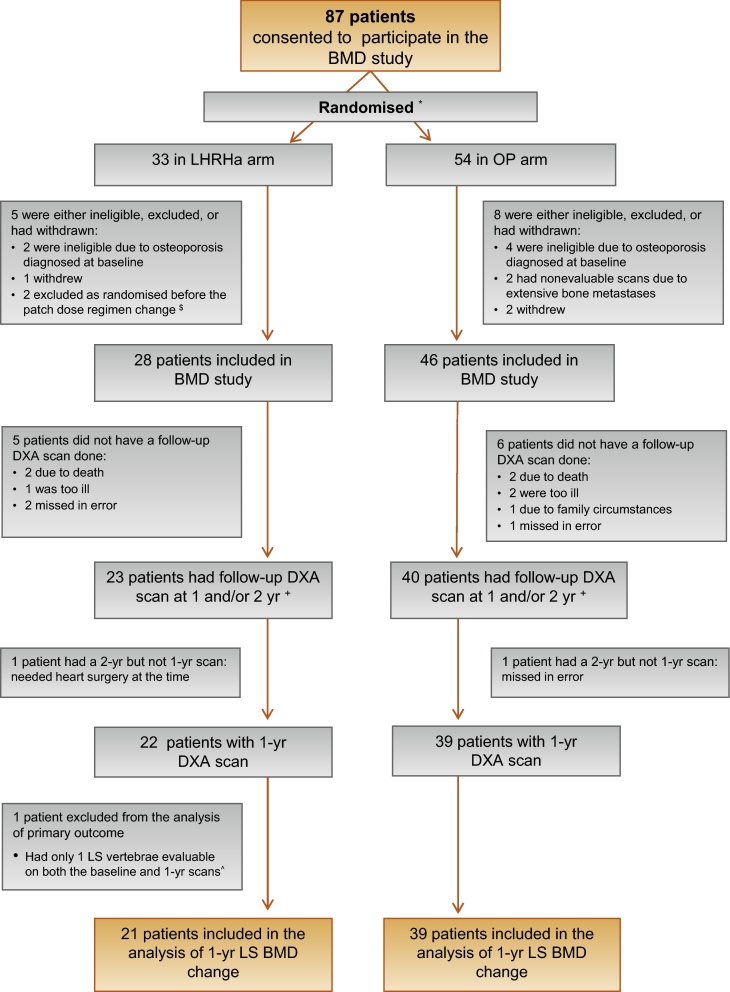
Flowchart of patients included in the analysis of change in lumbar spine bone mineral density at 1 yr from baseline (primary outcome measure). BMD = bone mineral density; DXA = dual-energy x-ray absorptiometry; LHRHa = luteinising hormone-releasing hormone agonists; LS = lumbar spine; OP = oestradiol patches. ^*^ The allocation ratio was 1:2 for LHRHa to OP before February 21, 2011, and 1:1 thereafter. ^$^ The patch dose regimen was increased in August 2007 [16]. ^+^ These patients contributed to at least one of the analyses on BMD change. ^^^ The main analysis of the primary outcome was restricted to patients with at least two evaluable LS vertebrae within L1–4 on *both* the baseline and 1-yr scans.

A total of 87 patients consented to participate in the study. Eleven were deemed ineligible after enrolment or did not proceed (Fig. 2); six were diagnosed with osteoporosis on the baseline scan, two had nonevaluable scans due to extensive bone metastases, and three withdrew. Another two were randomised before a patch dose change and so were excluded. Baseline clinical characteristics of the remaining 74 eligible patients were similar between the two arms (Table 1). Median age was 77 yr (interquartile range [IQR]: 73–80 yr), median baseline prostate-specific antigen (PSA) was 56 ng/ml (IQR: 30–112), and 43% (32 of 74) had metastatic disease.

At 3 mo, the proportion of patients with testosterone concentrations ≤1.7 nmol/l was 96% in both the LHRHa arm (26 of 27) and the OP arm (42 of 44), excluding two patients not adhering to the patch regimen (oestradiol <250 pmol/l) and one missing testosterone value. The corresponding proportion at 6 mo was LHRHa 85% (22 of 26) and OP 90% (37 of 41). Median oestradiol level at 3 mo was 70 pmol/l in the LHRHa arm (5th–95th centile range 19–114 pmol/l) and 685 pmol/l (350–1788 pmol/l) in the OP arm.

Overall, 63 patients with either 1-yr (*n* = 61) and/or 2-yr (*n* = 48) DXA scans were included in the analyses of change in BMD for at least one of the anatomic sites. The primary analysis of 1-yr change in LS BMD was based on 60 men (81% of 74 eligible patients) (Fig. 2). Three centres (*n* = 27 participants) used Hologic Discovery DXA machines, three used Lunar Prodigy (*n* = 19), and one used both Hologic Discovery (*n* = 11) and Hologic Delphi (*n* = 3). The proportion of men scanned using Hologic Discovery, the most commonly used machine, was 67% (14 of 21) in the LHRHa arm and 62% (24 of 39) in the OP arm. At the 1-yr scan, 19 of 21 LHRHa patients (90%) and 33 of 39 OP patients (85%) were reported to still be on the original allocated treatment only without additional anticancer therapy.

The mean 1-yr change in LS BMD was −0.021 g/cm^3^ for the LHRHa arm versus +0.069 g/cm^3^ for OP (*p* < 0.001). The corresponding mean percentage changes were −1.4% and +6.0%, respectively, with an estimated difference between arms of 6.7% (95% confidence interval [CI], 3.7–9.7) in favour of OP. As shown in Table 2, similar patterns with BMD decreasing in LHRHa patients and increasing in OP patients were seen at 1 yr for the right hip (difference between arms +3.8% [1.4–6.2%]; *p* = 0.003), left hip (+4.3% [1.7–6.9%]; *p* = 0.002), and for whole-body measurements (+2.5% [1.0–4.0%]; *p* = 0.002). Within the patch arm, there was no evidence of an association between serum oestradiol level and BMD change at any of the anatomic sites (data not shown).

The differences between arms remained in all predefined subgroup analyses, as shown in Table 3: patients still on original allocated treatment only, no bone metastases at baseline, scanned using Hologic Discovery machines, and all four L1–4 LS vertebrae evaluable at baseline and follow-up scans.

**Fig. 3:**
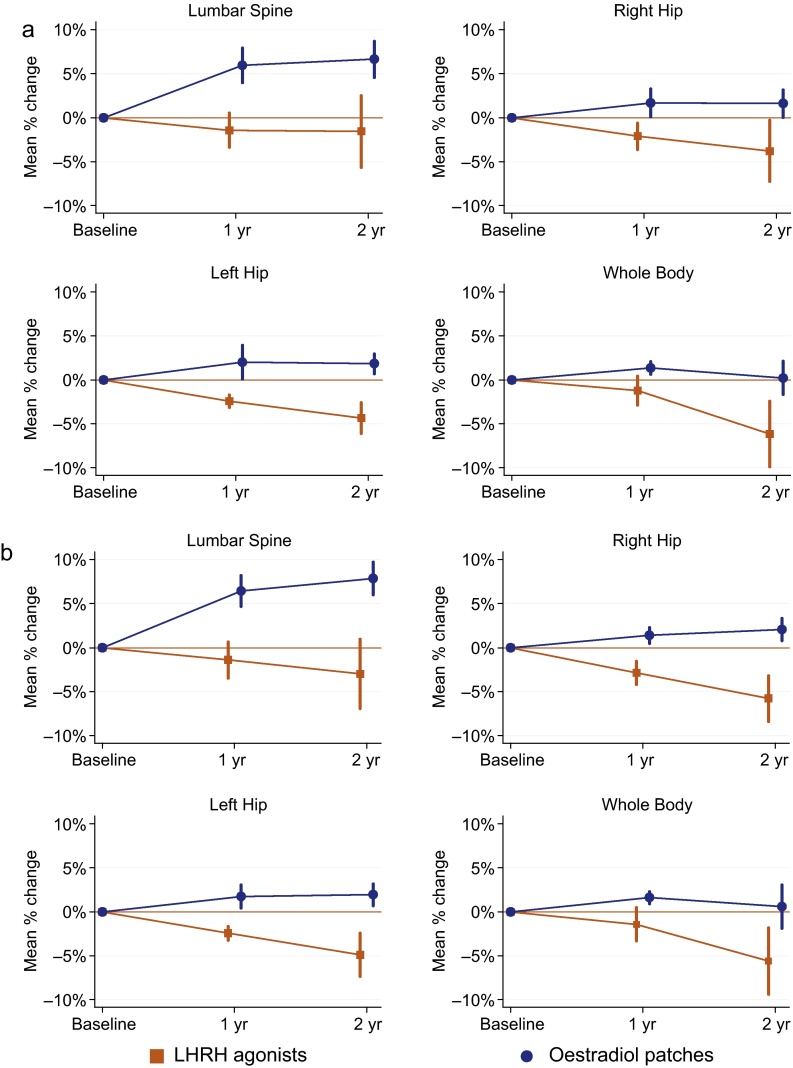
Mean percentage change (95% confidence interval) in bone mineral density at 1 and 2 yr from baseline by treatment arms. (a) All patients; (b) patients still on allocated treatment only (ie, patients who were still on allocated treatment at the time of the scan with no additional anticancer therapy, with those on oestradiol patch with oestradiol values <250 pmol/l assumed not to be adhering to the patch regimen). The analyses at 1 and 2 yr are based on different numbers of patients (see Table 2, Table 3). LHRH = luteinising hormone-releasing hormone.

Figure 3 shows the mean percentage BMD change over time. As expected, the greatest absolute difference between arms was seen at 2 yr among those still on allocated treatment only. Mean change in LS BMD was −0.047 g/cm^3^ (mean percentage change: −3.0%) for LHRHa and +0.088 g/cm^3^ (+7.9%) for OP (*p* < 0.001), with an estimated difference between arms of 9.3% (95% CI, 5.3–13.4). Similar trends for absolute BMD change are shown in Supplementary Figure 1.

## 4 Discussion

We report on the first randomised study to show that PCa patients treated with transdermal oestradiol for first-line hormonal therapy avoid the BMD loss associated with LHRHa administration. The effect of OP on BMD preservation was seen across different anatomic sites. As expected, the greatest absolute difference in BMD change between treatment arms was observed at 2 yr for those receiving only their allocated treatment for PCa during this period.

These findings are in line with the protective effect of oestradiol on bone health among postmenopausal women receiving oestrogen replacement therapy [20]. Our results are supported by two previous studies of parenteral oestradiol in PCa. First, a single-arm pilot study of 20 men treated with OP for newly diagnosed locally advanced or metastatic disease showed BMD increased at all measured sites over time [21]. Second, in the randomised Scandinavian Prostatic Cancer Group SPCG-5 trial (*n* = 910) evaluating parenteral oestradiol therapy in the form of intramuscular polyestriol phosphate (PEP), none of the patients in the PEP arm reported serious skeletal complications compared with 18 (mainly fractures) on a combined androgen blockade (with either LHRHa or orchidectomy) over a median follow-up of 11 yr [22]; however, BMD was not assessed during this study.

Our study, although relatively small, was sufficiently powered to detect the difference between arms we were expecting to observe, and it provided consistent evidence for the effect of OP across all prespecified analyses. It has certain limitations, however. First, fracture was not a secondary outcome because of the limited sample size and follow-up period, but data are being recorded within the main trial. Although a strong inverse relationship between BMD and fracture risk is known to exist, there are other determinants of bone strength and susceptibility to fracture [23]. Second, scans were not centrally reviewed, although sites were requested to perform standardised DXA procedures for quality control. In addition, different types of DXA machine were used across sites; however, individual patients were scanned with the same type of machine during follow-up, and machine type was further accounted for in statistical analysis.

The increasing use of LHRHa in PCa management has highlighted the need to better understand the nature and impact of ADT. Low testosterone results in loss of libido, erectile dysfunction, and decrease in muscle mass [24], [25]. Other toxicities associated with LHRHa such as osteoporosis, increased fracture risk, hot flashes, and dyslipidemia are thought to be due to oestradiol deficiency [25]. Oestradiol deficiency prolongs the life span of bone-resorptive osteoclasts, with the resulting imbalance between osteoclasts and bone-forming osteoblasts increasing the rate of bone thinning. This heightens the risk of fracture [26], [27], leading to increased morbidity and reduced quality of life [10], [28].

Potential strategies that have been suggested to mitigate the accelerated loss of BMD with LHRHa include the addition of calcium and vitamin D [6], bisphosphonates [29], selective oestrogen receptor modulators [6], and targeting the receptor activator of the nuclear factor kappa B ligand that blocks the maturation of osteoclasts [17]. However, recent meta-analyses found that calcium and vitamin D supplements have little impact on BMD, and there was insufficient and inconsistent evidence of an effect on fracture risk [30], [31], [32]. Both zoledronic acid and denosumab have been approved for reducing risk of skeletal-related events (SREs) in men with castration-resistant PCa and bone metastases. Interestingly, zoledronic acid was shown to be effective in preventing fractures and SREs in a meta-analysis of randomised trials of PCa patients by Serpa Neto et al, but not in the subsequent Cancer and Leukemia Group B (CALGB) 90202 trial which included men with castration-sensitive PCa and bone metastases [29], [33]. This may be due to differences in patient characteristics between studies. In particular, within the CALGB trial, zoledronic acid appeared to have an effect among men with an SRE before baseline but not those without. Furthermore, zoledronic acid was found to be inferior to denosumab for preventing SREs in a randomised trial of men with castration-resistant PCa [34]. Neither denosumab or zoledronic acid, however, was shown to improve OS [35], [36]. It is worth noting, in addition, that these various approaches involve adding further agents to LHRHa administration and could increase complexity for patients and health care costs. In contrast, transdermal oestradiol as a single agent produces castration levels of testosterone while appearing to preserve BMD.

The major concern about using oestradiol has been CVS toxicity [12]. However, early results from the PATCH trial [15] and previous work from Scandinavia on intramuscular PEP [37] suggest the excess CVS mortality seen with oral oestrogen is avoidable. In the initial cohort of 254 men in the PATCH trial with a median follow-up of 19 mo, the proportion of patients in the OP arm experiencing a CVS event (10.1%; 95% CI, 6.0–15.6) was relatively similar to that in the LHRHa arm (7.1%; 2.7–14.9), with half of the events assigned to men on OP occurring some time after treatment with the patches was stopped and LHRHa started. In comparison, oral oestrogen at 5 mg/d of DES was associated with a CVS mortality risk of 20% within the first 12 mo of treatment [12]. CVS outcomes are closely monitored within the ongoing PATCH trial (>900 patients recruited since 2006) and regularly reviewed by members of the IDMC who, to date, have recommended the trial continue. Another concern with utilising oestradiol therapy is gynaecomastia, experienced by approximately 75% of patients on patches within the trial, although it was generally mild with 10% having grade 3 events [15]. However, hot flashes, which can affect quality of life for men receiving ADT, are reduced with oestradiol therapy compared with LHRHa, and they were reported in 25% versus 56% of patients in the two respective arms during the first stage of PATCH [15]. Patients on OP appeared to develop more favourable blood glucose and lipid profiles [15].

## 5 Conclusions

This study identifies the first single agent that produces castration levels of testosterone comparable with LHRHa administration while mitigating BMD loss, thereby adding significantly to the evidence supporting further evaluation of parenteral oestradiol for treating PCa. The PATCH programme, to date, has also shown that transdermal oestradiol appears to avoid the CVS toxicity seen with oral oestrogen and potentially results in more favourable metabolic profiles than LHRHa [15]. Transdermal oestradiol may therefore be a potentially useful and cost-effective agent for ADT in systemic PCa, and the final results of the phase 3 trial will provide a full assessment of efficacy and toxicity. Validation of these BMD findings will also be required in larger cohorts. The development of alternative approaches to ADT potentially allows more personalised treatment for patients with PCa with improved toxicity profiles.

  ***Author contributions:*** Trinh Duong had full access to all the data in the study and takes responsibility for the integrity of the data and the accuracy of the data analysis.  

*Study concept and design:* Langley, Abel, Parmar, Kynaston, Alhasso.

*Acquisition of data:* Welland, Carpenter, Kynaston, Alhasso, Paez, Scrase, Robertson, Abel.

*Analysis and interpretation of data:* Langley, Kynaston, Alhasso, Duong, Paez, Jovic, Scrase, Robertson, Cafferty, Welland, Carpenter, Honeyfield, Abel, Stone, Parmar, Abel.

*Drafting of the manuscript:* Langley, Duong, Abel.

*Statistical analysis:* Jovic, Duong.

*Critical revision of the manuscript for important intellectual content:* Langley, Kynaston, Alhasso, Duong, Paez, Jovic, Scrase, Robertson, Cafferty, Welland, Carpenter, Honeyfield, Abel, Stone, Parmar, Abel.

*Obtaining funding:* Langley, Abel, Parmar, Kynaston, Alhasso.

*Administrative, technical, or material support:* Welland, Carpenter.

*Supervision:* Langley, Abel.

*Other* (specify): None.  

***Financial disclosures:*** Ruth E. Langley certifies that all conflicts of interest, including specific financial interests and relationships and affiliations relevant to the subject matter or materials discussed in the manuscript (eg, employment/affiliation, grants or funding, consultancies, honoraria, stock ownership or options, expert testimony, royalties, or patents filed, received, or pending), are the following: Ruth E. Langley has served as a consultant/advisor for Bayer and also received honoraria from the company.  

***Funding/Support and role of the sponsor:*** The PATCH trial is funded by Cancer Research UK via the Clinical Trials Advisory and Awards Committee (CTAAC), grant number C17093/A12443 (trial CRUK/06/001) and University College London (UCL). It is sponsored by Imperial College London. The funding sources and sponsor had no part in the study design, collection, analysis, or interpretation of the data, the writing of the manuscript, or the decision to submit.  

***Acknowledgments:*** We thank the National Institute for Health Research Cancer Research Network for staff support; all the patients who participated in the bone mineral density study (BMD) and their families; the research staff at the participating hospitals who contributed to the study (see appendix for list of participating hospitals); the PATCH Trial Management Group, Trial Steering Committee, and the Independent Data Monitoring Committee (see appendix for list of members). We also thank all previous and current members of the PATCH trial team including Suzanne Freeman, who contributed to the planning of the BMD study and programming of the statistical analysis, Ben Spittle, who was responsible for the trial coordination and data collection during the conduct of the BMD study, and Anna Bara, who oversees the practical running of the trial.

## Figures and Tables

**Table 1:** Baseline characteristics (*n* = 74)*

Characteristics	LHRHa (*n* = 28)		OP (*n* = 46)	
	*n*	%	*n*	%
Age, yr				
<70	4	14	9	19
70–79	15	54	24	52
≥80	9	32	13	28
Median (IQR)	77 (74–80)		76 (72–80)	
Smoking status				
Never smoked	12	43	18	39
Previous smoker	14	50	27	59
Current smoker	2	7	1	2
Metastatic disease	14	50	18	39
Bone metastases, those with metastatic disease, %	11	79	18	100
PSA, ng/ml				
<50	14	50	20	43
50 to <500	14	50	23	50
≥500	0	0	3	7
Median (IQR)	52 (25–91)		56 (30–127)	
Tumour status				
T2	0	0	2	4
T3	16	57	36	78
T4	10	36	6	13
TX	2	7	2	4
Gleason score at diagnosis				
4–6	4	15	7	16
7	6	22	20	44
8–10	17	63	18	40
Missing	1	–	1	*–*
Bone mineral density, median (IQR)				
Lumbar spine BMD, g/cm^3^	1.335 (1.100–1.451)		1.154 (1.078–1.283)	
Lumbar spine T-score	1.3 (0.1–2.6)		0.5 (−0.6–1.4)	
Left hip BMD, g/cm^3^	1.037 (0.947–1.168)		1.014 (0.921–1.075)	
Left hip T-score	−0.1 (−0.6 to 0.8)		−0.3 (−0.9 to 0.1)	
Right hip BMD, g/cm^3^	1.047 (0.982–1.162)		0.989 (0.912–1.079)	
Right hip T-score	0.1 (−0.6 to 0.8)		−0.5 (−1.0 to 0.2)	
Whole-body BMD, g/cm^3^	1.234 (1.182–1.416)		1.232 (1.148–1.361)	
Whole-body T-score	0.1 (−0.3 to 2.7)		0.3 (−0.8 to 1.9)	
Osteopenia**	8	29	17	37

BMD = bone mineral density; IQR = interquartile range; LHRHa = luteinising hormone-releasing hormone agonists; OP = oestradiol patches.

* The 60 patients included in the primary analysis of 1-yr lumbar spine BMD change had similar baseline characteristics as the overall group.

** Defined as T-score greater than −2.5 and less than or equal to −1.0 at any of the sites measured (patients with osteoporosis (T-score less than or equal to −2.5) were not eligible for the BMD study).

**Table 2:** Change in bone mineral density at 1 and 2 yr from baseline

Site	Arm	No. of patients	Mean absolute change, g/cm^3^ (SD)	Mean change, % (SD)	Difference between arms$, % (95% CI)	*p* value
At 1 yr						
Lumbar spine	LHRHa	21	−0.021 (0.057)	−1.4 (4.3)		
	OP	39	+0.069 (0.076)	+6.0 (6.1)	+6.7 (3.7–9.7)	<0.001
Right hip	LHRHa	21	−0.022 (0.033)	−2.1 (3.3)		
	OP	37	+0.016 (0.049)	+1.7 (4.8)	+3.8 (1.4–6.2)	0.003
Left hip	LHRHa	20	−0.026 (0.016)	−2.4 (1.5)		
	OP	34	+0.019 (0.055)	+2.0 (5.5)	+4.3 (1.7–6.9)	0.002
Whole body	LHRHa	17	−0.015 (0.043)	−1.2 (3.2)		
	OP	35	+0.017 (0.026)	+1.4 (2.1)	+2.5 (1.0–4.0)	0.002
At 2 yr						
Lumbar spine	LHRHa	12	−0.026 (0.086)	−1.6 (6.4)		
	OP	29	+0.077 (0.060)	+6.6 (5.4)	+8.1 (3.8–12.4)	0.001
Right hip	LHRHa	17	−0.040 (0.070)	−3.8 (6.8)		
	OP	29	+0.017 (0.044)	+1.6 (4.1)	+5.8 (2.3–9.3)	0.002
Left hip	LHRHa	16	−0.047 (0.036)	−4.3 (3.3)		
	OP	30	+0.018 (0.031)	+1.8 (3.0)	+6.4 (4.3–8.5)	<0.001
Whole body	LHRHa	15	−0.089 (0.106)	−6.2 (6.7)		
	OP	32	+0.002 (0.069)	+0.2 (5.3)	+6.0 (2.0–9.9)	0.006

CI = confidence interval; LHRHa = luteinising hormone-releasing hormone agonists; OP = oestradiol patches; SD = standard deviation.

$ Estimated using analysis of covariance models, as described in the Statistical analysis section.

**Table 3:** Change in bone mineral density from baseline: predefined subgroup analyses

Site	Arm	No. of patients	Mean absolute change, g/cm^3^ (SD)	Mean change, % (SD)	Difference between arms, % (95% CI)	*p* value
Patients on allocated treatment without additional anticancer therapy						
At 1 yr						
Lumbar spine	LHRHa	19	−0.021 (0.058)	−1.4 (4.3)		
	OP	33	+0.075 (0.059)	+6.5 (5.0)	+6.9 (4.2–9.7)	<0.001
Right hip	LHRHa	18	−0.030 (0.026)	−2.9 (2.6)		
	OP	31	+0.014 (0.026)	+1.4 (2.5)	+4.7 (3.2–6.2)	<0.001
Left hip	LHRHa	17	−0.026 (0.016)	−2.4 (1.5)		
	OP	28	+0.017 (0.034)	+1.7 (3.4)	+4.1 (2.2–6.1)	<0.001
Whole body	LHRHa	14	−0.019 (0.043)	−1.4 (3.3)		
	OP	29	+0.020 (0.022)	+1.6 (1.8)	+2.7 (1.1–4.3)	0.002
At 2 yr						
Lumbar spine	LHRHa	10	−0.047 (0.068)	−3.0 (5.5)		
	OP	23	+0.088 (0.049)	+7.9 (4.3)	+9.3 (5.3–3.4)	<0.001
Right hip	LHRHa	12	−0.061 (0.040)	−5.8 (4.1)		
	OP	22	+0.022 (0.030)	+2.1 (2.8)	+8.6 (6.1–11.0)	<0.001
Left hip	LHRHa	11	−0.053 (0.040)	−4.9 (3.7)		
	OP	22	+0.019 (0.029)	+1.9 (2.8)	+6.6 (4.1–9.2)	<0.001
Whole body	LHRHa	10	−0.078 (0.083)	−5.6 (5.3)		
	OP	24	+0.005 (0.077)	+0.6 (5.9)	+6.5 (2.1–10.9)	0.007
Patients without bone metastases at baseline: at 1 yr						
Lumbar spine	LHRHa	13	−0.043 (0.041)	−3.2 (2.8)		
	OP	25	+0.065 (0.066)	+5.8 (5.4)	+7.9 (4.9–10.9)	<0.001
Right hip	LHRHa	12	−0.021 (0.018)	−2.0 (1.8)		
	OP	23	+0.009 (0.025)	+0.9 (2.6)	+3.2 (1.4–4.9)	0.001
Left hip	LHRHa	12	−0.028 (0.018)	−2.7 (1.8)		
	OP	21	+0.014 (0.034)	+1.6 (3.5)	+4.0 (1.7–6.3)	0.002
Whole body	LHRHa	9	−0.021 (0.031)	−1.7 (2.4)		
	OP	22	+0.019 (0.021)	+1.6 (1.7)	+3.0 (1.5–4.5)	0.001
Patients scanned using Hologic Discovery machines: at 1 yr *						
Lumbar spine	LHRHa	14	−0.008 (0.042)	−0.7 (3.8)		
	OP	24	+0.069 (0.069)	+6.0 (5.6)	+6.7 (3.5–10.0)	<0.001
Right hip	LHRHa	14	−0.016 (0.030)	−1.6 (2.9)		
	OP	22	+0.022 (0.059)	+2.2 (5.7)	+3.7 (0.3–7.0)	0.04
Left hip	LHRHa	15	−0.024 (0.018)	−2.3 (1.7)		
	OP	24	+0.023 (0.060)	+2.3 (5.9)	+4.7 (1.5–7.9)	0.007
Whole body	LHRHa	13	−0.017 (0.042)	−1.5 (2.9)		
	OP	23	+0.017 (0.024)	+1.3 (1.9)	+2.8 (1.2–4.5)	0.001
Patients with all four L1–L4 lumbar spine vertebrae evaluable: at 1 yr **						
Lumbar spine	LHRHa	19	−0.024 (0.049)	−1.7 (3.8)		
	OP	28	+0.063 (0.074)	+5.5 (6.0)	+6.0% (2.9–9.1)	<0.001

CI = confidence interval; LHRHa = luteinising hormone-releasing hormone agonists; OP = oestradiol patches; SD = standard deviation.

* The most commonly used dual-energy x-ray absorptiometry machine in the study.

** On both the baseline and 1-yr scans (because bone mineral density varies with lumbar spine vertebrae).

## Appendix

Hospitals participating in the BMD study: Charing Cross Hospital, Freeman Hospital, Glasgow Beatson West of Scotland Cancer Centre, Ipswich Hospital, Scarborough General Hospital, University Hospital of Wales, New Victoria Hospital/Southern General Hospital (Glasgow).

PATCH Trial Committees:

Trial Management Group (in alphabetical order): Paul Abel (chief investigator), Abdulla A. Alhasso, Anna Bara, Robin Carpenter, Noel W. Clarke, David Dearnaley, Trinh Duong, Ian F. Godsland, Gordana Jovic, Ruth E. Langley (co-principal investigator), Howard G. Kynaston, Roger Kockelbergh, Mahesh K.B. Parmar, Michael Philips (patient representative), Stuart D. Rosen, Andrew Welland.

Trial Steering Committee: David Guthrie (chair), John Chester, and Richard Cowan

Independent Data Monitoring Committee: Peter Hoskin (chair), Philip Smith, and Laurence Collette.
