# Supplementary material for: A Randomised Comparison Evaluating Changes in Bone Mineral Density in Advanced Prostate Cancer: Luteinising Hormone-releasing Hormone Agonists Versus Transdermal Oestradiol
Source: Eur Urol. 2016 Jun;69(6):1016–25. doi: 10.1016/j.eururo.2015.11.030 (PMC4854173; doi:10.1016/j.eururo.2015.11.030)
Supplement: Supplementary file 1 [file mmc1.ppt]

## Slide 1
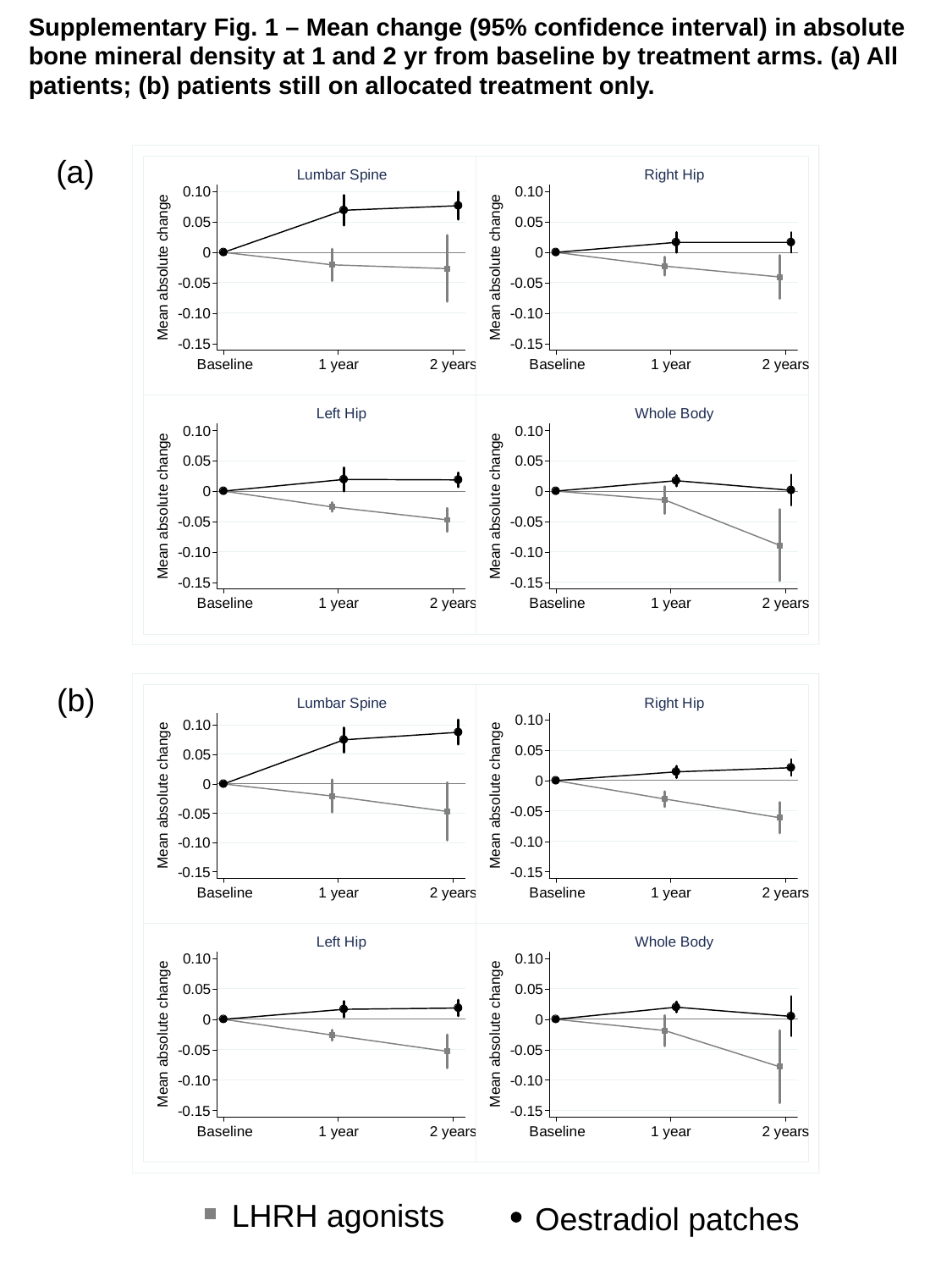

Supplementary Fig. 1 – Mean change (95% confidence interval) in absolute bone mineral density at 1 and 2 yr from baseline by treatment arms. (a) All patients; (b) patients still on allocated treatment only.
(a)
(b)
 LHRH agonists
 Oestradiol patches
